# Unlocking the potential of the UK 100,000 Genomes Project—lessons learned from analysis of the “Congenital Malformations caused by Ciliopathies” cohort

**DOI:** 10.1002/ajmg.c.31965

**Published:** 2022-03-15

**Authors:** Sunayna Best, Chris F. Inglehearn, Christopher M. Watson, Carmel Toomes, Gabrielle Wheway, Colin A. Johnson

**Affiliations:** ^1^ Division of Molecular Medicine Leeds Institute of Medical Research at St. James's, University of Leeds, St. James's University Hospital Leeds UK; ^2^ Yorkshire Regional Genetics Service Leeds UK; ^3^ North East and Yorkshire Genomic Laboratory Hub, Central Lab St. James's University Hospital Leeds UK; ^4^ University Hospital Southampton NHS Foundation Trust Southampton UK; ^5^ Faculty of Medicine, Human Development and Health University of Southampton Southampton UK

We reviewed sequencing, variant and clinical data from patients recruited to the “Congenital Malformations caused by Ciliopathies” (CMC) cohort of the UK 100,000 Genomes Project (100K) (Best et al., 2021).^1^ By using domain‐specific knowledge of ciliopathy genetics (Reiter & Leroux, 2017; Wheway et al., 2019), and examining variants in non‐ciliopathy disease genes, we were able to identify potentially causative variants beyond those reported by the triaging process implemented by Genomics England (GEL, the company set up to run 100K). As a result, we increased diagnoses from the 27/83 (32.5%) that were reported by GEL, to 43/83 (51.8%). During this work, we experienced several difficulties in accessing and working with the data and observed several limitations with the currently available datasets. Here, we review these issues, suggest ways in which 100K data could be made more accessible and utilized more fully for patient benefit, and propose lessons that can be learned for future large‐scale human genomics studies.

The issues are grouped into four broad categories: those relating to the clinical information available for recruited individuals; issues relating to the triaging and prioritization process for variants (so‐called “tiering”); difficulties experienced using the secure GEL research environment; and difficulties in reporting pertinent research findings back to recruiting clinicians.

## 1 PHENOTYPING ISSUES

In the early stages of recruitment to 100K, recruiters were required to comply with strict entry criteria. These included pre‐screening of the key genes or gene panels relevant to the participant's condition, the recruitment of parent‐child trios and adherence to a complex, time‐consuming process for the uploading of Human Phenotype Ontology (HPO) terms. However, pressure to recruit from busy NHS clinics led to relaxation of requirements for pre‐screening and trio recruitment, and frequently resulted in sparse HPO term usage, with patient phenotypes often described using only one or two terms from one organ system. The choice of organ system may have reflected the interests and expertise of the recruiting clinician: for instance, many participants in the CMC cohort were recruited under solely vision‐related terms such as rod‐cone dystrophy, with limited or absent information about extra‐ocular, syndromic features. As a result, the relevance of HPO terms varies across the cohort, ranging from accurate and highly informative to unhelpful or even misleading. Additional data from longitudinal patient records are accessible using the “Participant Explorer” tool, but these are available only in a proportion of cases, are of variable quality and are not collated in a form that can be readily used for phenotype‐genotype correlation and variant prioritization.

The accuracy of HPO descriptions has a direct impact on diagnostic success. 100K was configured to communicate results only from one or more virtual panels of genes that are defined in the GEL PanelApp database as relevant to a participant's suspected condition, based on entered HPO terms (although there is also ethical approval for broader variant screening on a research basis). The selection of gene panels is therefore largely dependent on the HPO descriptions. Thus, inappropriate HPO descriptions will inevitably lead to inappropriate gene panel selection and therefore to missed diagnoses because the correct disease gene(s) have not been analyzed. For example, a participant in the “epilepsy plus other features” category, with keratoconus and epilepsy as the entered HPO terms, was found to have bi‐allelic pathogenic *CEP290* variants. In a reverse phenotyping approach following contact with the recruiting clinician, it emerged that this participant had key ophthalmological features that were not entered during recruitment to 100K, comprising a formal diagnosis of Leber Congenital Amaurosis.

## 2 TIERING ISSUES

The GEL tiering system prioritizes variants for analysis by regional NHS diagnostic laboratories. Clinical assessment is only expected for prioritized Tier 1 and 2 single nucleotide variants (SNVs) or Tier A structural variants, which are provided in a report. Tiered variants are primarily limited to those variants affecting coding sequences and splice donor or acceptor sites. These are rare protein damaging (Tier 1) or protein altering (Tier 2) variants in genes on selected panel(s) in which the allelic state matches the known mode of inheritance for the gene and disorder, and segregates with disease where familial sequence data is available. Copy number variants and structural variants have been classified Tier A (>10 kb in appropriate PanelApp genes) or Tier Null in cases recruited toward the end of the project, but these have not yet been systematically analyzed in the whole cohort.

Rare SNVs in genes not on the selected panel(s) are classed as Tier 3. These include variants known or predicted to be pathogenic but not in a relevant PanelApp gene, or in a relevant gene but considered insufficient to explain the phenotype, such as a heterozygous variant in a gene implicated in recessive disease. All other variants are un‐tiered (although white‐listing of known pathogenic variants is an area of active development). Tier 3 and un‐tiered variants are not inspected routinely by NHS diagnostic labs, and left to external researchers to consider more fully, if at all. In our own work (Best et al., 2021), we identified 11/83 probands (13.3%) with research molecular diagnoses with at least one variant outside of tiers 1 and 2. Five tier 3 variants and 12 untiered variants contribute to the diagnoses for these 11 participants. Furthermore, no attempt has yet been made to prioritize less obvious splicing defects using SpliceAI (Jaganathan et al., 2019) or a similar program, or to analyze variants in intronic regions. One obvious limitation of reporting based on these partial analyses is that many recessive alleles appear monoallelic because the second allele is a structural, splice or intronic variant missed by the current GEL pipeline. These single recessive pathogenic alleles in relevant PanelApp genes will then be classed as Tier 3 and not prioritized for analysis because they alone cannot explain the participant's phenotype.

Anecdotally, we understand that some participants were recruited because a diagnostic laboratory had previously identified one variant in a relevant recessive gene, and the referring clinician anticipated that genome analysis would reveal the second. Instead, the eventual report was negative, lacking even the known variant, leading to confusion for clinicians and clinical scientists. Given the ever‐increasing demand for genetic testing, there seems little likelihood that NHS laboratories will have the operational flexibility to reassess these data in response to improvements in the GEL variant detection pipeline. In practice, therefore, although participants have a whole genome sequence, variant identification is typically no better than a targeted gene panel analysis.

## 3 USING THE GEL SECURE RESEARCH ENVIRONMENT

Given the limitations of the variant identification and triaging carried out by GEL itself, any further screening is dependent on individual researchers revisiting the data on a research basis. Our experience of the GEL secure research environment is that it can be a frustrating and uninviting area within which to work. Service interruption is not infrequent and can lead to work disruption and data loss. Scripts must be self‐contained for security reasons and must be security checked before importing, meaning users tend to work with and adapt what is already there rather than importing alternative tools and pipelines that are more fit for purpose. Opportunities for training are limited, meaning the aspiring genomics researcher is often dependent on generous collaborators who are already familiar with the research environment and are willing to share their skills and code. Use of the Linux command line is required for several investigative strategies within the GEL research environment, which is unfamiliar and intimidating to many inexperienced clinicians and scientists and requires significant time investment to master. The lack of a forum for script sharing, advice and learning from others seems a significant omission. An MSc program in Genomic Medicine was intended to address this deficit, but many of the funded programs completed before the data was released, missing an opportunity for hands‐on training within the GEL secure research environment. We accept that many of these issues arise from the need to protect patient data, which will in turn limit the scope for changes to the GEL research environment. Nevertheless, these difficulties have the effect of making training and collaboration more difficult and are a further disincentive to those wishing to work with 100K data. That many still do is a testament to the huge potential research value of this resource, but any efforts to make it more accessible could significantly enhance exploitation and patient benefit.

## 4 REPORTING PROBLEMS

We encountered significant problems disseminating identified diagnoses to recruiting clinicians, which limited the returning of results to patients and publication of findings. Reporting of research findings must be carried out through the 100,000 Genomes Airlock system using the “Researcher Identified Potential Diagnosis” and “Clinician Contact” process. The researcher submits their findings to this system using a request form, which is sent to the recruiting clinician, who remains anonymous unless and until they choose to respond. In our experience, the response rate is less than 20%. This may reflect the time that has elapsed since recruitment (2013–2018), meaning that some clinicians may have moved post.

Such a low response rate is another major obstacle to research on 100K cohorts. Researchers can publish un‐identifiable overview findings without involving recruiting clinicians, but must obtain consent from clinicians and participants before publishing detailed individual phenotypic data. Limited engagement by recruiting clinicians at best restricts, but may even prevent, the publication of findings, a major driver of research activity. Furthermore, researchers are unable to assess detailed phenotyping data or to obtain additional clinical samples from patients or relatives that could help segregation testing or functional analyses of variants. These issues limit researchers' opportunities to interpret the pathogenicity of variants, further reducing opportunities to benefit patients by making a definitive molecular diagnosis, and to publish.

## 5 FUTURE USE OF 100K DATA

During the period 2016–2018, many clinicians were encouraged to recruit to this project in preference to local clinical exome screening on the basis that it was a more comprehensive test. Screening to date has fallen well short of that promise, and despite the predicted 1 to 2‐year turnaround, reports have still not been issued for some patients. Nevertheless, the 100K dataset remains a powerful resource of immense value to patients, clinicians and researchers, both in the UK and globally. Whole genome sequence data can be revisited indefinitely, reducing the need for expensive and sometimes invasive serial tests frequently required in the “diagnostic odyssey” for patients with genetic diseases.

We suggest that a more agnostic approach to gene panel selection, like that used by the Deciphering Developmental Disorders project, rather than one driven narrowly by HPO term usage, would be beneficial. This approach would permit analysis of additional panels of genes with broadly overlapping phenotype ranges if an answer is not obtained from the relevant PanelApp gene panel, or if phenotyping data are not well documented. Reanalysis should also include approaches to identify variants likely to alter splicing and likely pathogenic structural variants, for example using SpliceAI and the SVRare suite of programs (Yu et al., 2021). This broader approach could identify “second hits” in relevant genes that appear to be monoallelic for tiered variants, and remain refractory to current strategies. Additionally, increasing accessibility for research teams around the world and reporting of new research‐based findings could reap further benefits for patients and clinicians. Updating the security software could make it easier to access and use, especially for research‐minded clinicians, without compromising security risks.

To derive maximum benefit from these efforts, lines of communication between researchers and clinicians should be improved. This may require an overhaul and update of the database of recruiting clinician contact details held by GEL, with new contacts established when clinical responsibility changes hands. In our experience, when the recruiting clinician did respond, the information they supplied proved invaluable in confirming molecular diagnoses. Often, many additional clinical features which had not been listed in the entered HPO terms were provided, which facilitated more accurate genotype‐phenotype correlation and greater diagnostic confidence. All new findings, whether generated through reanalysis by GEL or by researchers applying domain‐specific knowledge, would still need accredited diagnostic confirmation, so additional staff and resources for service testing are also essential.

As well as addressing issues within the existing study, the experience of those involved in 100K can inform future large‐scale human genomics studies. The use of HPO terms to describe and define phenotypes, if applied effectively, could facilitate an AI‐based, phenotype‐informed variant prioritization approach. A simple, comprehensively applied HPO term entry system could significantly enhance the value of any future human genome resource.

In summary, the ciliopathies provide an exemplar group of disorders that illustrate both the challenges and opportunities of working with 100K datasets (Best et al., 2021; Wheway et al., 2019). 100K remains an immensely valuable clinical and scientific resource with huge potential for patient benefit, but that benefit has not yet been fully realized. There is an urgent need for re‐evaluation of the data in light of improvements in genome interpretation technologies. Additional understanding could also be gained from research activity, which would benefit from efforts to simplify access, and train and support more researchers in using the data.

## CONFLICT OF INTEREST

None.

## Data Availability

Data sharing is not applicable to this article as no new data were created or analyzed in this study.

## ENDNOTE

^1^
Published 2021 with data from Main Program Release 11 (dated December 17, 2020). At that time, 16/83 of the “Congenital Malformations caused by Ciliopathies” cohort (19.3%) did not have a complete GMC exit questionnaire.
